# Early computed tomography coronary angiography in patients with suspected acute coronary syndrome: randomised controlled trial

**DOI:** 10.1136/bmj.n2106

**Published:** 2021-09-29

**Authors:** Alasdair J Gray, Carl Roobottom, Jason E Smith, Steve Goodacre, Katherine Oatey, Rachel O’Brien, Robert F Storey, Nick Curzen, Liza Keating, Attila Kardos, Dirk Felmeden, Robert J Lee, Praveen Thokala, Steff C Lewis, David E Newby, RAPID-CTCA Investigators, Tim Coats, Kenneth Archibald, Graham Bell, Russell Bull, Gerry McCann, Tarun Mittal, Rodney Mycock, James Rudd, Carrol Gamble, Simon Carley, Simon Padley, Nick Mills, Andrew Chapman, Anoop Shah, Mark Jones, Fergus Perks, Gareth Morgan-Hughes, Vikram Raju, Kym Luke, Andrew Kinnon, Ajay Yerramasu, Robert Huggett, Sudantha Bulugahapitiya, Jehangir Din, Andrew Mitchell, Anne Scott, Anna Beattie, Khalid Alfakih, Adrian Brady, Hefin Jones, Derek Connolly, Ronak Rajani, Rangasamy Muthusamy, Simon Smith, Abdel-Rahman Saif-El-Dean, Ben Holloway, Ansuman Saha, Matthias Schmitt, Christopher Travill, Ceri Davies, Tim Harris, Will Roberts, Patrick Donnelly, Justin Carter, John Irving, Chris Vorwerk, Ash Basu, Jason Dungu, Elisa McAlindon, Sandeep Hothi, David Rosewarne, Arivalagan Bapusamy, Jonathan Watt, Claire McGroarty, Graham McKillop,, Polly Black, Caroline Blackstock, Julia Grahamslaw, Collette Keanie, Margaret MacLeod, Siobhan McLaughlin, Alyson Phillips, Gillian Ritchie, Kirsty Simpson, Miranda Odam, Michelle Williams, Ewan Pirie, Janet Summerside, Gordon Truong, Kirsty Weston, Jennifer Wooton, Sarah Bird, Peter Brown, Hridesh Chatha, Alan Fletcher, Catherine Hill, Shery Mofidi, Hasan Qayyum, Judith Sugden, Anna Wilson, Alison Jeffrey, Memory Mwadeyi, Rosalyn Squire, Peter Wafer, Lesley Archer, Lisa Felmeden, Guy Gribbin, Sarah Harrison, Debbie Hughes, Philip Keeling, Ian Mahy, Allison Summerhayes, Justine Sutton, Abdullah Yonis, Susan Fowler, Amanda McGregor, Karen Grey, Tom Hartley, David Szapiro, Lorraine Dinnel, Dennis Sandeman, Julie Dean, Amy Pugh, Parminder Bhuie, James Briggs, Claire Burnett, Abby Gandy, Nicola Jacques, Sarah MacGill, Archie Speirs, Niamh Tolan, Craig Atkinson, Mark Kon, Carita Krannila, Manitha Thomas, Russell Bull, Stephanie Horler, Nicki Lakeman, Jane McLeod, Sara Nix, Sue Thomas, Daniel Ahlert, Christopher Edmond, Christopher Hare, Kelly Anne Kinsella, Jessica Langtree, James Speakman, Ranjit Thomas, Gillian Donaldson, Fiona Hall, Terry Fairbairn, Christopher Rofe, Jennifer Adams-Hall, Ange Bailey, Kris Bailey, Leslie Bremner, Ifti Haq, Angela Phillipson, Saroj David, Osman Najam, Samia Pilgrim, Claire Adams, Ammani Brown, Andrew Dougherty, Ailsa Geddes, Karen Lang, David Lowe, Ross MacDuff, Lorraine McGregor, Giles Roditi, Susan Thornton, Joyce Triscott, Felicia Adjei, Antoanela Colda, Caitlin Chapman, Veronica Edgell, Michael Fell, Laszlo Halmai, Aarzoo Khan, John Northfield, Cheryl Padilla-Harris, Mike Pashler, Gill Richie, Diane Scaletta, Sarah–Beth Sunderland, Joanne Turner, Lois Vickery, Sonya Walia, Felicity Williams, Lynn Wren, Nicola Wright, Holly Maguire, Resti Varquez, Anthony D’Sa, Vinoda Sharma, Ashley Turner, Megan Bell, Giulia Benedetti, Kirsty Gibson, Sze Mun Mak, Rebecca Preston, Amy Raynsford, Ruth Sanchez-Vidal, Susan Biggins, Kathryn Dixon, Peter Kraut, Mwada Lawan, Victoria Murray, Tom Mwambingu, Rachel Walker, Carol Weston, Roo Byrom-Goulthorp, Michael Darby, Eunice Ikongo, Annette Johnstone, Alan Lin, Melanie Mcginlay, Tania Albutt, Vicky Dawson, Claire Dowling, Karen Isaacs, Cheyanne Kaila, Gareth Lewis, Nicky Mortimer, Sunitha San, Kelly Tabor, Kealy Wright, Riaz Ahmed, Sally Collins, Sarah Davies, Nokukhanya Ndlovu, Ausami Abbas, Alison Calver, Simon Corbett, Peter Cowburn, Andrew Flett, Huon Gray, Stephen Harden, Paul Haydock, Michael Mahmoudi, John Paisey, Charles Peebles, Drew Rakhit, John Rawlins, Paul Roberts, Benoy Shah, James Shambrook, Iain Simpson, Rohit Sirohi, Wagas Ullah, Katharine Vedwan, James Wilkinson, Arthur Yue, Sarra Giannopoulou, Melanie Greaves, Stephen McGlynn, Chris Miller, Akhila Muthuswamy, Lindsay Murray, Anie Nicholas, Matthias Schmitt, Susan Gent, Nafisa Hussain, Raine Astin-Chamberlain, Ben Bloom, Olivia Bolton, Dan Martin, Lyrics Noba, Georgia Norman, Shelley Page, Helen Power, Imogen Skene, David Smith, Jon Walters, Angela Doughty, Elaine Byng-Hollander, Helen Routledge, Leah Hammond, Jayne Hutchinson, Stephanie Kelly, Susan Regan, Aileen Smith, Julie Gray, Sarah Purvis, Pam Race, Christine Almaden-Boyle, Kim Bissett, Carol Blues, Jackie Duff, Scot Dundas, Shirley Fawcett, Graeme Houston, Emma Hutchison, Debbie Letham, Ann Mackintosh, Laura Meach, Laura Jayne Queripcz, Alan Webster, Julian Atchley, Zoe Daly, Kat Ellinor, Richard Cowell, Helen Craddock, Rachel Hughes, Lynda Sackett, Victoria Saul, Fiona Smith, Jane Stockport, Clare Watkins, Edward Barden, Jackie Colnet, Swamy Gedeza, Laura Hoskin, Lauren Kittridge, Gracie Maloney, Claire McCormick, Anne Nicholson, Stacey Pepper, Joanne Riches, Annaliza Sevillano, Vincent Amoah, Stacey Aulton, Arivalagan Bapusamy, Victoria Cottam, Stella Metherell, Sarah Milgate, Elizabeth Radford, David Rosewarne, Andy Smallwood, Charlotte Barr, Jonathan Broadie, David Eason, Ing-Marie Logie, Debbie McDonald, Laura O’Keeffe, Donna Patience, Lesley Patience, Faheem Ahmad, Nicola Baxter, Ammani Brown, John Byrne, Damien Collison, Tracey Hopkins, Hayley King, David Lowe, Evonne McLennan, Giles Roditi, David Stobo, Mark Wilson, Rosie Woodward, Ruth Armstrong, Julia Boyd, David Buchanan, Christine Campbell, Ronnie Harkess, Lynsey Milne, Lumine Na, Phillip Rayson, Aryelly Rodriguez, Pamela Sinclair, Lorraine Smith, Michelle Stevens, Tony Wackett, Allan Walker, Christopher White

**Affiliations:** 1 University of Edinburgh, Edinburgh, UK; 2 Royal Infirmary of Edinburgh, NHS Lothian, Edinburgh, UK; 3 University Hospitals Plymouth NHS Trust, Plymouth, UK; 4 University of Plymouth, Plymouth, UK; 5 University of Sheffield, Sheffield, UK; 6 University of Southampton, Southampton, UK; 7 Royal Berkshire NHS Foundation Trust, Reading, UK; 8 Milton Keynes University Hospital NHS Foundation Trust, Milton Keynes, UK; 9 University of Buckingham, Buckingham, UK; 10 Torbay and South Devon NHS Foundation Trust, Torquay, UK

## Abstract

**Objectives:**

To establish if the use of early computed tomography (CT) coronary angiography improves one year clinical outcomes in patients presenting to the emergency department with acute chest pain and at intermediate risk of acute coronary syndrome and subsequent clinical events.

**Design:**

Randomised controlled trial.

**Setting:**

37 hospitals in the UK.

**Participants:**

Adults with suspected or a provisional diagnosis of acute coronary syndrome and one or more of previous coronary heart disease, raised levels of cardiac troponin, or abnormal electrocardiogram.

**Interventions:**

Early CT coronary angiography and standard of care compared with standard of care only.

**Main outcome measures:**

Primary endpoint was all cause death or subsequent type 1 or 4b myocardial infarction at one year.

**Results:**

Between 23 March 2015 and 27 June 2019, 1748 participants (mean age 62 years (standard deviation 13), 64% men, mean global registry of acute coronary events (GRACE) score 115 (standard deviation 35)) were randomised to receive early CT coronary angiography (n=877) or standard of care only (n=871). Median time from randomisation to CT coronary angiography was 4.2 (interquartile range 1.6-21.6) hours. The primary endpoint occurred in 51 (5.8%) participants randomised to CT coronary angiography and 53 (6.1%) participants who received standard of care only (adjusted hazard ratio 0.91 (95% confidence interval 0.62 to 1.35), P=0.65). Invasive coronary angiography was performed in 474 (54.0%) participants randomised to CT coronary angiography and 530 (60.8%) participants who received standard of care only (adjusted hazard ratio 0.81 (0.72 to 0.92), P=0.001). There were no overall differences in coronary revascularisation, use of drug treatment for acute coronary syndrome, or subsequent preventive treatments between the two groups. Early CT coronary angiography was associated with a slightly longer time in hospital (median increase 0.21 (95% confidence interval 0.05 to 0.40) days from a median hospital stay of 2.0 to 2.2 days).

**Conclusions:**

In intermediate risk patients with acute chest pain and suspected acute coronary syndrome, early CT coronary angiography did not alter overall coronary therapeutic interventions or one year clinical outcomes, but reduced rates of invasive angiography while modestly increasing length of hospital stay. These findings do not support the routine use of early CT coronary angiography in intermediate risk patients with acute chest pain and suspected acute coronary syndrome.

**Trial registration:**

ISRCTN19102565, NCT02284191.

## Introduction

Chest pain is one of the commonest complaints in patients presenting to the emergency department.^1^ ^2^ These patients are evaluated for acute coronary syndrome with urgent diagnostic investigation so that those with acute myocardial infarction or at risk of acute myocardial infarction receive appropriate treatment promptly. Lack of clarity on the optimal diagnostic pathways, however, has led to the development of risk scores and the use of numerous functional and anatomical testing options to investigate for underlying coronary artery disease.^3^ ^4^ ^5^ ^6^ ^7^ Recent guidelines have proposed that patients at low or intermediate risk of coronary artery disease should undergo observation and further testing if acute coronary syndrome is suspected.^5^ ^6^ ^7^

Several trials have explored the role of computed tomography (CT) coronary angiography in low risk patients presenting to the emergency department with chest pain.^8^ ^9^ ^10^ ^11^ The trials showed that CT coronary angiography increased rates of discharge from hospital and shortened length of stay compared with usual care. These findings are likely to apply only where usual care for patients at low risk involves high rates of admission and investigation. Participants were at low risk of coronary heart disease (<10%) and consequently had low rates of cardiovascular events (0.1-0.8%), leading some to suggest that non-invasive testing was unnecessary and only clinical evaluation was required.^12^ ^13^

Although recommended in guidelines^5^ ^6^ ^7^ as a potential strategy for subsequent investigation, the use of early CT coronary angiography in patients presenting with acute chest pain, who are at intermediate risk of acute coronary syndrome and subsequent clinical events, has not been investigated or established. This strategy could identify patients who would benefit from more rapid and appropriate therapeutic interventions, thus improving clinical outcomes.^14^ ^15^ In those patients without disease, CT coronary angiography might reduce the need for invasive coronary angiography, shorten hospital stay, and avoid repeated admissions to hospital. If CT coronary angiography does not influence investigations, treatments, and outcomes of patients, however, it might increase the cost and risk without any clinical benefit. Our trial aimed to investigate the effect of early CT coronary angiography on the management and outcome of patients with suspected or a provisional diagnosis of acute coronary syndrome, presenting to the emergency department.

## Methods

### Trial design

We performed a prospective, randomised, open, blinded endpoint, parallel group clinical effectiveness trial in 37 hospitals across the UK. The trial protocol has been reported previously.^16^

### Participants and randomisation

From March 2015 to June 2019, adults with symptoms of suspected acute coronary syndrome or those with a provisional diagnosis of acute coronary syndrome and one or more of previous coronary heart disease, raised levels of cardiac troponin, or abnormal electrocardiogram (ECG), were recruited in the emergency department or hospital admission facilities. Table S1 lists the full eligibility criteria. Written informed consent was provided by all participants who were recruited early after admission to hospital and randomised 1:1, grouped by site, and with variable block sizes (4-8), to early CT coronary angiography with standard of care or standard of care only, with a web based randomisation service to ensure that allocation to the group was concealed.

### Trial intervention

ECG gated calcium score and contrast enhanced CT coronary angiograms were conducted with ≥64 slice scanners. All centres were encouraged to use techniques to reduce radiation and heart rate, and to use sublingual glyceryl trinitrate. CT coronary angiograms were reported according to the Society of Cardiovascular CT guidelines^17^ and the American Heart Association coronary artery segment model.^18^ Severity of disease was categorised as no coronary artery disease (cross sectional stenosis <10%), mild non-obstructive disease (cross sectional stenosis 10-49%), moderate non-obstructive disease (cross sectional stenosis 50-70%), or obstructive coronary artery disease (cross sectional stenosis >70% or >50% in the left main stem). Standard of care was at the discretion of the attending clinician although guidance on treatment based on the CT coronary angiogram results was provided to all sites (table S2).

### Outcomes

An independent clinical endpoint committee, blinded to the trial intervention, adjudicated the trial primary outcomes. The primary endpoint was time to the first event of all cause death or subsequent non-fatal type 1 (spontaneous) or type 4b (related to stent thrombosis) myocardial infarction at one year. Myocardial infarction was defined according to the 2012 universal definition of myocardial infarction.^19^ Key secondary endpoints were cause of death (coronary heart disease or cardiovascular death) and subsequent myocardial infarction. Table S3 describes the trial endpoints. Several changes were made to the trial protocol, including refining the secondary endpoints during the trial (table S4). The final and most substantial changes were made to align the endpoints with the SCOT-HEART (Scottish Computed Tomography of the HEART) trial to allow direct comparisons (protocol version 7, 24 February 2020).^14^ ^15^

### Study power and sample size

The original sample size calculation was estimated from a one year death or subsequent myocardial infarction rate of about 20%.^20^ At 90% power and a two sided P value of <0.05, 2424 evaluable patients were required to detect a reduction in death or subsequent myocardial infarction from 20% to 15% at one year. After review of the first 716 participants, the overall event rate was 6.8% (95% confidence interval 5.2% to 8.9%). Given this lower than anticipated 12 month event rate and the much slower rates of recruitment, we consulted with the trial funder and trial steering committee and recalculated the sample size, with blinded data, to 1735 participants to detect a 3.4% absolute risk reduction at a revised primary event rate of 6.8% with 80% power and a two sided P value <0.05. These reductions in relative effect size have been reported in previous meta-analyses of randomised controlled trials of low risk patients presenting to the emergency department.^11^

### Statistical analysis

The trial was reported on an intention to treat basis. Descriptive data are presented as number (percentage), mean (standard deviation), or median (interquartile range). The primary outcome was defined as time to the first event of all cause death or subsequent non-fatal myocardial infarction type 1 or 4b and was analysed with Cox proportional hazards regression adjusted for study site (used to stratify the randomisation), global registry of acute coronary events (GRACE) score,^21^ and previous coronary heart disease. Study site was included as a random effect and GRACE score with a restricted cubic spline. For patients who left the trial before reaching the primary outcome, time was censored at the last contact date. 

Secondary outcomes were analysed with Cox proportional hazards regression for time-to-event outcomes and logistic regression for binary outcomes, and proportional odds logistic regression for ordinal outcomes, adjusted in the same way as the primary outcome. For the primary outcome and key secondary outcomes, we used the fixed sequence method to test for significance; statistical significance was if the outcome, and all prior outcomes listed, had a two sided P value of <0.05.^22^ Subgroup analyses were performed on the primary outcome for age, sex, GRACE score, previous coronary heart disease, raised levels of cardiac troponin, abnormal ECG, and onsite invasive coronary angiography facilities, with treatment-subgroup interaction in the Cox model. The proportional hazards assumption was checked for the time-to-event outcomes, and we found evidence of non-proportional hazards only for the outcome of non-invasive coronary artery disease or myocardial ischaemia testing. The analysis was performed with SAS software (version 9.4, SAS System for Windows).

### Patient and public involvement

The trial team liaised with members of the Sheffield Emergency Care Forum (a patient and public representative group that provides independent advice on emergency care related research) during the grant application where they reviewed the proposal and provided advice on study design, patient procedures, and ethical issues, which helped inform the final submission and subsequent study design. The forum was also consulted during the development of the trial information (patient information letters, consents, general practitioner letter), and the documents were amended to incorporate their feedback, which helped improve the usability of the documents. During trial delivery, patient representatives participated in the trial steering committee and were involved in the oversight of the trial throughout its duration. The forum provided valuable feedback about the patient perspective throughout the trial, which helped guide the decision making of the trial team. The Sheffield Emergency Care Forum helped develop the plain English summary for the final funder report. Members of the forum will help develop material to allow us to disseminate the trial findings to the public.

## Results

### Participants

**Fig 1:**
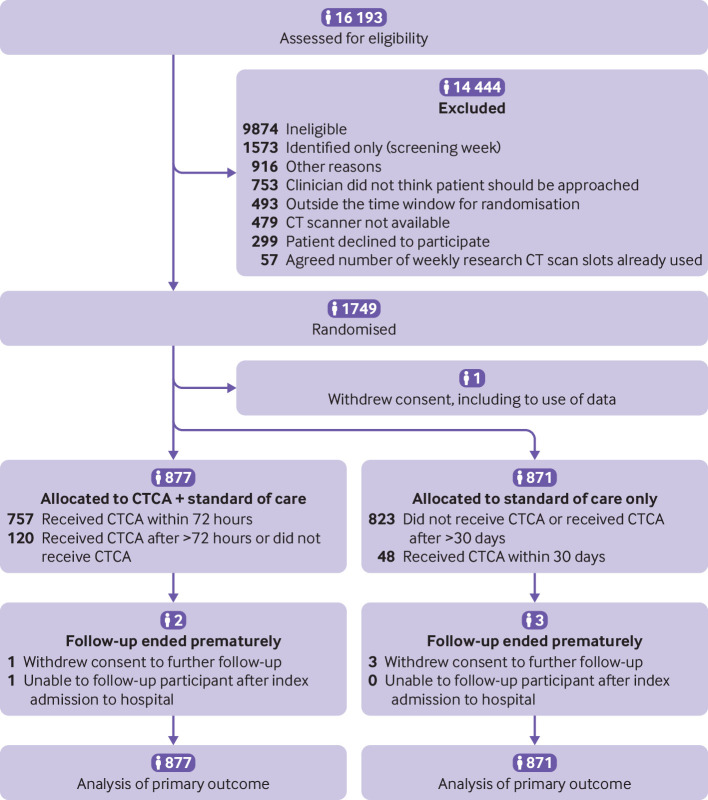
Flowchart of recruitment and participants. CT=computed tomography; CTCA=computed tomography coronary angiography

Between 23 March 2015 and 27 June 2019, we recruited 1749 patients, with 1748 participants available for analysis (fig 1). Recruitment stopped when the revised recruitment target was reached in June 2019, and the trial ended when the planned follow-up was subsequently completed in July 2020. The mean age of participants was 61.6 (12.6) years, and 1114 (64%) were men. At recruitment, 601 (34%) participants had previous coronary heart disease, 1004 (57%) had raised levels of cardiac troponin, and 1064 (61%) had an abnormal ECG. Chest pain was the primary complaint in 1549 (89%) participants, with 857 (49%) diagnosed as having an acute coronary syndrome (myocardial infarction or unstable angina) at discharge from their index admission to hospital (table S5). The mean GRACE score was 115 (35) with 410 (23%) participants having a GRACE score >140. Baseline characteristics and follow-up were similar for the two groups (table 1 and fig 1).

**Table 1:** Baseline patient characteristics

	CT coronary angiography and standard of care	Standard of care only	Overall
No of participants	877	871	1748
Age (mean (SD))	61.9 (12.2)	61.2 (13.0)	61.6 (12.6)
Male sex	564 (64)	550 (63)	1114 (64)
Previous coronary heart disease	302 (34)	299 (34)	601 (34)
Raised levels of cardiac troponin	492 (56)	512 (59)	1004 (57)
Abnormal electrocardiogram	549 (63)	515 (59)	1064 (61)
GRACE score:			
Mean (SD)	115 (36)	114 (34)	115 (35)
Low risk (<109)	390 (44)	384 (44)	774 (44)
Intermediate risk (109-140)	268 (31)	296 (34)	564 (32)
High risk (>140)	219 (25)	191 (22)	410 (23)
Recruited at hospital with onsite invasive coronary angiography facilities	644 (73)	641 (74)	1285 (74)
Presenting complaint:*			
Chest pain	776 (89)	773 (89)	1549 (89)
Shortness of breath	35 (4)	31 (4)	66 (4)
Palpitation	17 (2)	15 (2)	32 (2)
Collapse	10 (1)	10 (1)	20 (1)
Other	38 (4)	42 (5)	80 (5)
Cardiovascular risk factors:			
Diabetes mellitus	153 (17)	165 (19)	318 (18)
Hypertension	413 (47)	404 (46)	817 (47)
Hyperlipidaemia	358 (41)	336 (39)	694 (40)
Current or ex-smoker	530 (60)	531 (61)	1061 (61)
Family history†	269 (31)	270 (31)	539 (31)
Medical history:			
Myocardial infarction‡	180 (21)	171 (20)	351 (20)
Previous coronary angiography	222 (25)	214 (25)	436 (25)
Previous PCI§	115 (13)	123 (14)	238 (14)
Previous CABG surgery¶	52 (6)	48 (6)	100 (6)
Cerebrovascular disease	35 (4)	38 (4)	73 (4)
Peripheral vascular disease	27 (3)	28 (3)	55 (3)
Preventive treatment:			
Aspirin treatment	203 (23)	212 (24)	415 (24)
Statin treatment	283 (32)	298 (34)	581 (33)
ACE inhibitor or ARB treatment	217 (25)	216 (25)	433 (25)

Data are number (%) unless stated otherwise.

CT=computed tomography; GRACE=global registry of acute coronary events; PCI=percutaneous coronary intervention; CABG=coronary artery bypass graft; ACE=angiotensin converting enzyme; ARB=angiotensin receptor blocker; SD=standard deviation.

* Missing data: one participant in CT coronary angiography and standard of care arm.

† Missing data: four participants in CT coronary angiography and standard of care arm and one participant in standard of care only arm.

‡ Missing data: one participant in standard of care only arm.

§ Missing data: three participants in standard of care only arm.

¶ Missing data: one participant in standard of care only arm.

### Intervention

Of those patients randomised to CT coronary angiography, 767 (87.5%) underwent CT coronary angiography; table S6 provides the reasons for non-adherence. Median time from randomisation to CT coronary angiography was 4.2 (interquartile range 1.6-21.6) hours (fig S1). The CT coronary angiography scan was of diagnostic quality in 700 (91.3%) patients, with a median effective radiation dose of 5.8 (3.5-10.3) mSv (0.026 mSv/mGy cm conversion factor) and was associated with four related adverse events (one readmission with a possibly related non-cardiac condition and three non-serious adverse events related to the intravenous cannula). In the standard of care arm, 48 (5.5%) participants had a CT coronary angiogram within 30 days of randomisation. CT coronary angiography identified normal coronary arteries in 178 (23%) patients, non-obstructive disease in 222 (29%), and obstructive disease in 359 (47%) (table 2).

**Table 2:** Clinical characteristics and subsequent management according to CT coronary angiogram findings in participants randomised to CT coronary angiography

	Normal coronary arteries	Non-obstructive coronary artery disease	Obstructive coronary artery disease
No of participants	178	222	359
Age (mean (SD))	54.9 (12.4)	63.3 (11.4)	64.0 (11.6)
Male sex	67 (38)	132 (59)	279 (78)
Previous coronary heart disease	27 (15)	85 (38)	144 (40)
Raised levels of cardiac troponin	69 (39)	104 (47)	249 (69)
Abnormal electrocardiogram	114 (64)	129 (58)	231 (64)
GRACE score:			
Low risk (<109)	125 (70)	107 (48)	108 (30)
Intermediate risk (109-140)	34 (19)	64 (29)	141 (39)
High risk (>140)	19 (11)	51 (23)	110 (31)
Onsite coronary angiography	139 (78)	163 (73)	254 (71)
Invasive coronary angiogram performed	25 (14)	83 (37)	289 (81)
Acute coronary syndrome treatment*	105 (59)	145 (65)	271 (75)
Coronary revascularisation	7 (4)	26 (12)	222 (62)
Preventive treatments†	65 (37)	143 (64)	274 (76)

Data are number (%) unless stated otherwise.

CT=computed tomography; GRACE=global registry of acute coronary events; SD=standard deviation.

* Treatment for acute coronary syndrome prescribed during index admission to hospital.

† Primary or secondary prevention treatment started, stopped, or dose altered during index admission to hospital.

Eight participants had CT coronary angiography from which the coronary arteries were not classified as normal, non-obstructive disease, or obstructive disease, and these participants have not been included.

### Primary and key secondary outcomes

**Fig 2:**
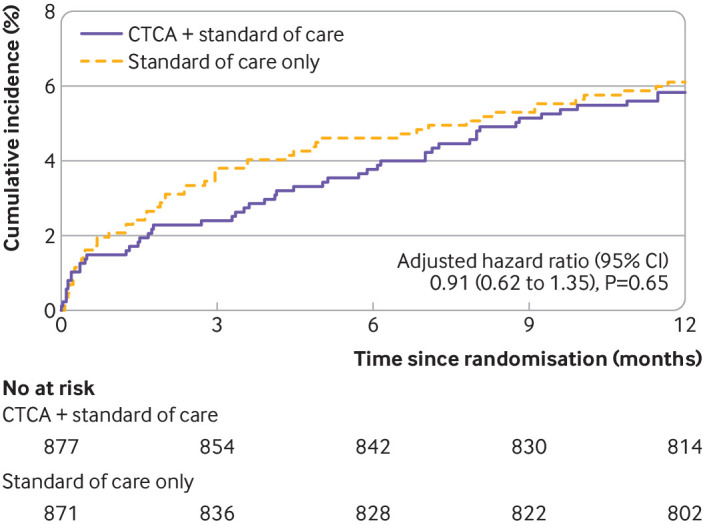
Cumulative incidence of primary endpoint of one year all cause death or non-fatal myocardial infarction (type 1 or 4b). CTCA=computed tomography coronary angiography

**Fig 3:**
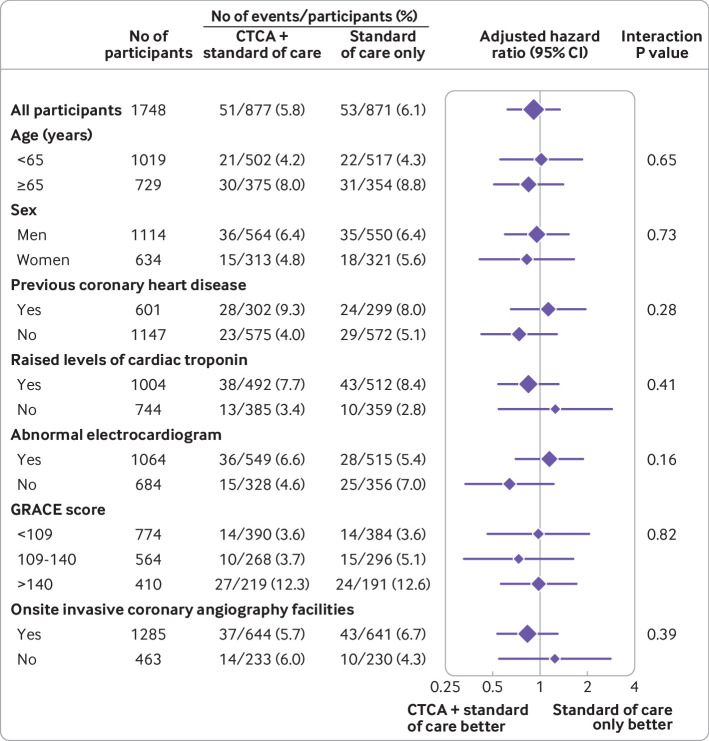
Prespecified subgroup analyses of one year all cause death or non-fatal myocardial infarction (type 1 or 4b). GRACE=global registry of acute coronary events; CTCA=computed tomography coronary angiography

The primary outcome of all cause death or non-fatal myocardial infarction (type 1 or 4b) at one year occurred in 51 (5.8%) of the 877 participants in the early CT coronary angiography arm and in 53 (6.1%) of the 871 participants in the standard of care arm (adjusted hazard ratio 0.91 (95% confidence interval 0.62 to 1.35), P=0.65; fig 2 and table 3). For the prespecified subgroup analysis for the primary outcome, we found no significant heterogeneity for any comparison (fig 3). We found no evidence of a difference between allocated treatment arms for any of the key secondary outcomes (table 3). Other clinical outcomes (death from coronary heart disease or subsequent myocardial infarction type 1 or 4b; subsequent myocardial infarction type 1 or 4b; and non-cardiovascular death) were also similar (table S7).

**Table 3:** Primary and key secondary outcomes

	CT computed angiography and standard of care (n=877)	Standard of care only (n=871)	Estimate	Hazard ratio (95% CI)	P value*
**Primary outcome**					
All cause death or non-fatal myocardial infarction (type 1 or 4b)	51 (5.8)	53 (6.1)	Unadjusted	0.95 (0.65 to 1.40)	0.79
			Adjusted	0.91 (0.62 to 1.35)	0.65
**Secondary outcomes **					
Death from coronary heart disease or non-fatal myocardial infarction	47 (5.4)	45 (5.2)	Unadjusted	1.03 (0.69 to 1.55)	0.88
			Adjusted	1.02 (0.67 to 1.53)	0.94
Death from cardiovascular disease or non-fatal myocardial infarction	48 (5.5)	46 (5.3)	Unadjusted	1.03 (0.69 to 1.54)	0.88
			Adjusted	1.01 (0.68 to 1.52)	0.95
Non-fatal myocardial infarction	39 (4.4)	40 (4.6)	Unadjusted	0.96 (0.62 to 1.50)	0.87
			Adjusted	0.95 (0.61 to 1.47)	0.81
Death from coronary heart disease	11 (1.3)	6 (0.7)	Unadjusted	1.82 (0.67 to 4.92)	0.24
			Adjusted	1.78 (0.66 to 4.82)	0.26
Death from cardiovascular death	12 (1.4)	8 (0.9)	Unadjusted	1.49 (0.61 to 3.64)	0.38
			Adjusted	1.39 (0.57 to 3.42)	0.47
All cause death	19 (2.2)	17 (2.0)	Unadjusted	1.11 (0.58 to 2.13)	0.76
			Adjusted	1.03 (0.53 to 1.99)	0.94

Data are number (%) unless stated otherwise. Adjusted hazard ratios are from models adjusting for study site, GRACE (global registry of acute coronary events) score, and previous coronary heart disease.

CT=computed tomography.

* Nominal P values provided for secondary outcomes given the primary outcome was not statistically significant.

### Processes of care and other outcomes

Satisfaction of participants (rated excellent or very good on a five point Likert scale) was higher in the early CT coronary angiography arm than in the standard of care arm (adjusted odds ratio 1.25 (95% confidence interval 1.02 to 1.53), P=0.03; table 4). The attending clinician reported increased diagnostic certainty after CT coronary angiography: mean increase of 1.4 (2.2) on a 0-10 scale (from 7.1 to 8.5, with 10 being most certain).

**Table 4:** Medical treatment and other outcomes

	CT coronary angiography and standard of care (n=877)	Standard of care only (n=871)	Estimate	Odds ratio (95% CI)	P value
Inhospital medical treatment for acute coronary syndrome	595 (67.8)	580 (66.6)	Unadjusted	1.06 (0.87 to 1.29)	0.58
			Adjusted	1.06 (0.85 to 1.32)	0.63
Change in preventive treatment	554 (63.2)	539 (61.9)	Unadjusted	1.06 (0.87 to 1.28)	0.58
			Adjusted	1.07 (0.87 to 1.32)	0.52
Preventive treatment:					
Started	526 (60.0)	509 (58.4)	—	—	—
Stopped	71 (8.1)	61 (7.0)	—	—	—
Dose changed	91 (10.4)	100 (11.5)	—	—	—
Participant satisfaction with service received:*	—	—	Unadjusted†	1.23 (1.01 to 1.51)	0.04
			Adjusted†	1.25 (1.02 to 1.53)	0.03
Poor	10 (1.5)	7 (1.1)	—	—	—
Fair	21 (3.0)	28 (4.5)	—	—	—
Good	84 (12.2)	92 (14.7)	—	—	—
Very good	226 (32.8)	215 (34.3)	—	—	—
Excellent	348 (50.5)	285 (45.5)	—	—	—

Data are number (%) unless stated otherwise.

CT=computed tomography.

* n=689 for CT coronary angiography and standard of care; n=627 for standard of care only.

† Unadjusted and adjusted odds ratios are from post hoc analysis with proportional odds logistic regression model to estimate the common odds ratio for higher levels of satisfaction.

**Fig 4:**
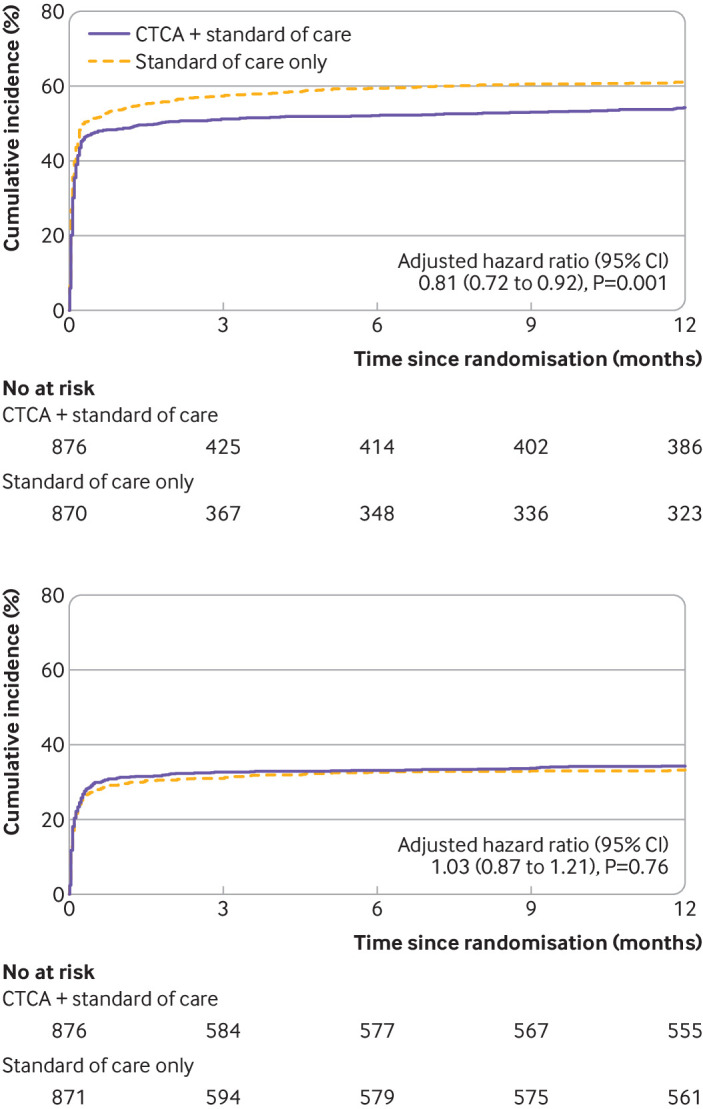
Cumulative incidence of invasive coronary angiography (top) and coronary revascularisation (bottom). One participant in each of the treatment arms had unknown date of invasive coronary angiography and one participant in CTCA + standard of care arm had unknown date of coronary revascularisation and therefore these participants were not included in the time-to-event analyses. CTCA=computed tomography coronary angiography

**Fig 5:**
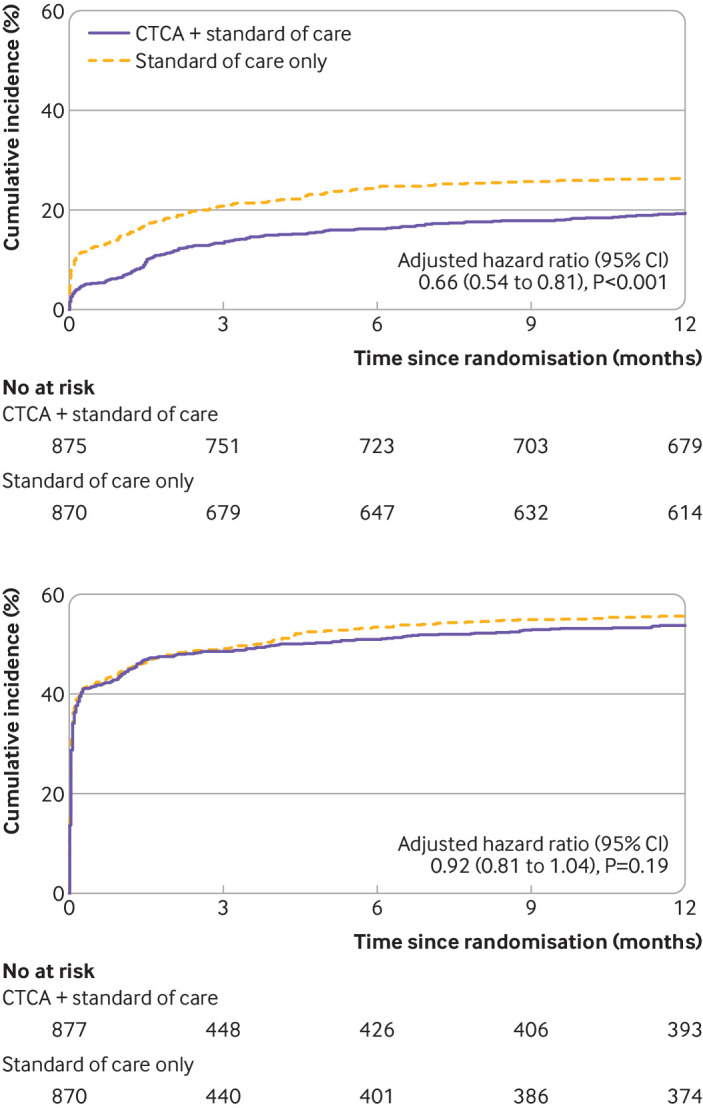
Cumulative incidence of non-invasive coronary artery disease or myocardial ischaemia testing (top) and other non-invasive cardiac investigations (bottom) from post hoc analysis. Evidence of non-proportional hazards for non-invasive coronary artery disease or myocardial ischaemia testing was found, and adjusted hazard ratio was reported as an estimate indicative of the average effect. One participant in CTCA + standard of care arm had unknown date of exercise electrocardiogram and one participant in each treatment arm had unknown date of cardiac magnetic resonance angiogram and therefore these participants were not included in the time-to-event analysis for non-invasive coronary artery disease or myocardial ischaemia testing. One participant in standard of care only arm had unknown date of echocardiogram and therefore this participant was not included in the time-to-event analysis for other non-invasive cardiac investigations. CTCA=computed tomography coronary angiography

Fewer participants in the CT coronary angiography arm received invasive coronary angiography: 474 (54.0%) compared with 530 (60.8%) in the standard of care arm (adjusted hazard ratio 0.81 (95% confidence interval 0.72 to 0.92), P=0.001; table 5 and fig 4). Despite fewer invasive coronary angiograms in the CT coronary angiography arm, we found no evidence of a difference in the rates of coronary revascularisation by group (adjusted hazard ratio 1.03 (0.87 to 1.21), P=0.76; table 5 and fig 4). In a post hoc analysis, subsequent rates of non-invasive testing for coronary artery disease and myocardial ischaemia were lower in the CT coronary angiography arm: 170 (19.4%) compared with 228 (26.2%) in the standard of care arm (adjusted hazard ratio 0.66, 0.54 to 0.81, P<0.001), although for other cardiac investigations (echocardiography and rhythm monitoring) no evidence of a difference was found (adjusted hazard ratio 0.92, 0.81 to 1.04, P=0.19; table 5 and fig 5).

**Table 5:** Processes of care outcomes over 12 months

	CT coronary angiography and standard of care (n=877)	Standard of care only (n=871)	Estimate	Hazard ratio (95% CI)	P value
Investigations					
Invasive coronary angiography	474 (54.0)	530 (60.8)	Unadjusted	0.83 (0.74 to 0.94)	0.004
			Adjusted	0.81 (0.72 to 0.92)	0.001
Non-invasive coronary artery disease or myocardial ischaemia testing (post hoc analysis)*	170 (19.4)	228 (26.2)	Unadjusted‡	0.69 (0.56 to 0.84)	<0.001
			Adjusted‡	0.66 (0.54 to 0.81)	<0.001
Other non-invasive cardiac investigation (post hoc analysis)†	470 (53.6)	484 (55.6)	Unadjusted	0.95 (0.83 to 1.07)	0.40
			Adjusted	0.92 (0.81 to 1.04)	0.19
**Coronary revascularisation**					
Coronary revascularisation	300 (34.2)	288 (33.1)	Unadjusted	1.03 (0.88 to 1.22)	0.68
			Adjusted	1.03 (0.87 to 1.21)	0.76
Percutaneous coronary intervention	260 (29.6)	240 (27.6)	Unadjusted	1.08 (0.90 to 1.28)	0.42
			Adjusted	1.08 (0.90 to 1.28)	0.42
Coronary artery bypass graft surgery	52 (5.9)	55 (6.3)	Unadjusted	0.94 (0.64 to 1.37)	0.73
			Adjusted	0.91 (0.62 to 1.33)	0.63
**Recurrent hospital attendance**					
Re-presentation or recurrent admission to hospital with suspected acute coronary syndrome or recurrent chest pain	138 (15.7)	130 (14.9)	Unadjusted	1.06 (0.83 to 1.34)	0.65
			Adjusted	1.06 (0.83 to 1.34)	0.66

Data are number (%) unless stated otherwise.

CT=computed tomography.

* Stress echocardiogram, stress magnetic resonance imaging, stress nuclear myocardial perfusion imaging, exercise electrocardiogram, cardiac magnetic resonance imaging or angiogram, or CT coronary angiogram not as trial intervention.

† Echocardiogram or electrocardiographic rhythm monitoring.

‡ Evidence of non-proportional hazards was found, and unadjusted and adjusted hazard ratios are reported as estimates indicative of the average effect.

Drug treatments for acute coronary syndrome were prescribed in hospital for 595 (67.8%) participants in the CT coronary angiography arm and in 580 (66.6%) in the standard of care arm (adjusted odds ratio 1.06 (95% confidence interval 0.85 to 1.32), P=0.63). At hospital discharge, the change in prescription for preventive treatments was similar: 554 (63.2%) participants in the CT coronary angiography arm compared with 539 (61.9%) in the standard of care arm (1.07 (0.87 to 1.32), P=0.52, table 4).

Median length of hospital stay was longer in the CT coronary angiography arm: 2.2 (interquartile range 1.1-4.1) days compared with 2.0 (1.0-3.8) days in the standard of care arm (Hodges-Lehmann estimator of location shift, 0.21 (95% confidence interval 0.05 to 0.40) days; P=0.009). We found no difference in the discharge diagnosis of acute coronary syndrome (myocardial infarction or unstable angina): 50.2% in the CT coronary angiography arm compared with 47.9% in the standard of care only arm (table S5). Chest pain symptoms, re-attendance at hospital, and quality of life at one year were unchanged by the trial intervention (table 5, table S8, table S9, and fig S2).

## Discussion

### Principal findings

This multicentre, randomised controlled trial aimed to establish the clinical effectiveness of early CT coronary angiography in the management and outcome of patients presenting to the emergency department with suspected acute coronary syndrome or with a provisional diagnosis of acute coronary syndrome. We found no evidence that CT coronary angiography had an effect on the one year rate of death or subsequent non-fatal myocardial infarction. Also, no evidence of benefit for any of the prespecified subgroup analyses or key secondary outcomes, or length of stay, was found. We conclude that the routine use of early CT coronary angiography is not an appropriate strategy to reduce one year clinical events in intermediate risk patients with acute chest pain.

We recruited a large population of patients with suspected acute coronary syndrome or those with a provisional diagnosis of acute coronary syndrome who had a spectrum of risk, reflected in the range of GRACE scores. Our trial population was equally composed of those who had or did not have a final diagnosis of acute coronary syndrome, and those who had or did not have obstructive coronary artery disease, a population that was truly representative of patients with an intermediate level of risk. 

The use of early CT coronary angiography had no effect on rates of treatments for acute coronary syndrome, with similar rates of use of drug treatment for acute coronary syndrome, coronary revascularisation, and preventive treatments at discharge from hospital. These findings are likely to reflect the high sensitivity of current clinical assessment, combining testing for cardiac troponin with a 12 lead ECG. Current practice therefore provided limited opportunity for CT coronary angiography to identify patients with unrecognised acute coronary syndrome caused by coronary heart disease. Moreover, for CT coronary angiography to improve adverse coronary events, it would need to alter the management of patients. These changes have been seen in previous studies of patients with stable chest pain where CT coronary angiography improved the detection of unrecognised coronary artery disease, increasing the use of preventive treatments and coronary revascularisation. These changes in management were associated with reduced rates of subsequent death from coronary heart disease or non-fatal myocardial infarction.^14^ ^15^ In our population of patients with acute chest pain and relatively high rates of invasive coronary angiography and coronary revascularisation, we found no such changes. We might have recruited too many patients who were at high risk of disease and therefore were candidates for inpatient invasive coronary angiography. However, more than three quarters of participants had a low or intermediate GRACE risk score, and less than 50% had obstructive coronary artery disease. In our prespecified subgroup analyses, no evidence of heterogeneity of effect for the primary outcome according to the level of risk was found. These subgroups are the focus of contemporary guidelines,^5^ ^6^ ^7^ which attempt to define intermediate risk groups suitable for observation and additional testing, including CT coronary angiography. We could not show an effect of CT coronary angiography on one year clinical outcomes in any of these subgroups or risk categories.

### Benefits of early CT coronary angiography

Early CT coronary angiography was associated with some benefits. Patients valued the use of CT coronary angiography, possibly reflecting a quicker evaluation of their clinical condition and the enhanced diagnostic certainty of the attending clinician. CT coronary angiography was also associated with a reduction in invasive coronary angiography, contrasting with previous trials of acute chest pain where CT coronary angiography was associated with increased rates of invasive angiography.^8^ ^9^ ^10^ ^11^ ^23^ This disparity likely reflects differences in trial populations, especially the baseline risk and prevalence of coronary heart disease. 

We recruited intermediate risk patients who were found to have a high prevalence of disease (50-75% with coronary artery disease) in contrast with the low prevalence (<10%) in previous studies.^8^ ^9^ ^10^ ^11^ Moreover, recent adoption of high sensitivity troponin testing has supported earlier clinical decision making^3^ ^4^ ^5^ ^6^ ^7^ but has misidentified many patients who do not have myocardial infarction.^24^ The strength of CT coronary angiography is its high negative predictive value for coronary artery disease, which applies to all risk groups, including those patients with non-ST segment elevation acute coronary syndrome.^25^ We found that 40-50% of patients with normal or non-obstructive coronary artery disease had raised levels of cardiac troponin. Our finding of a 19% relative reduction in the hazard for invasive coronary angiography likely reflects the exclusion of obstructive coronary artery disease, thereby avoiding unnecessary invasive coronary angiography, especially in patients with raised levels of cardiac troponin not attributable to myocardial infarction or obstructive coronary artery disease. 

The reduction in invasive coronary angiography was not associated with differences in overall rates of coronary revascularisation or clinical events, suggesting excellent diagnostic accuracy and continued appropriate coronary revascularisation for those with obstructive coronary artery disease. Also, CT coronary angiography was associated with a reduction in the need for further downstream testing for ischaemia, underlying its use in diagnosis and the associated improved diagnostic certainty reported by clinicians.

### Comparison with other studies

Previous trials^8^ ^9^ ^10^ have used CT coronary angiography for early and safe discharge of low risk patients from the emergency department. These studies were mostly designed to look at length of stay, but meta-analyses^11^ ^23^ indicated that early CT coronary angiography was not only associated with shorter lengths of stay but also with increased rates of invasive angiography and coronary revascularisation. These findings were not replicated in a multicentre trial of 500 low risk patients where rates of coronary angiography, revascularisation, and clinical events were unchanged by CT coronary angiography.^26^ Our trial provided data for those at higher risk than these previous studies and showed that the influence of CT coronary angiography was different in this population of patients. We found that CT coronary angiography can avoid unnecessary coronary angiography without affecting rates of coronary revascularisation. For hospitals with no ready access to invasive coronary angiography facilities, CT coronary angiography could therefore be a useful approach to identify those patients who do not require transfer to another hospital or further evaluation with invasive coronary angiography.

Previous comparisons of early CT coronary angiography compared with invasive coronary angiography have shown that up to 80% of invasive angiography can be avoided in patients with a low prevalence of coronary artery disease.^27^ ^28^ We found a more modest reduction in angiography in our trial, reflecting the greater prevalence of obstructive coronary heart disease in our study population. Also, many patients underwent invasive angiography and, for these participants, CT coronary angiography increased exposure to radiation and contrast, although median effective radiation doses were low, and no serious adverse reactions were found. Although undertaking CT coronary angiography in acutely unwell patients can be challenging, we found that more than 90% of the CT coronary angiograms were of diagnostic quality, comparing favourably with previous studies of CT coronary angiography in patients with stable^14^ ^15^ and acute chest pain.^25^ Also, the CT coronary angiograms clearly identified those with and without obstructive coronary artery disease, and this finding is reflected in the differences in the selection of those who later underwent invasive coronary angiography and coronary revascularisation. Our findings are consistent with previous studies^25^ ^29^ and suggest that CT coronary angiography could safely be used as a first line investigation where onsite invasive angiography is not available or there is a delay in availability, or in patients where the clinical diagnosis of myocardial infarction is uncertain.

### Limitations of the trial

The trial had some limitations. Open trials have the potential for bias. By design, however, the primary endpoint was assessed by an independent clinical endpoint committee who were blinded to group allocation. Also, the trial intervention did not affect the overall rates of treatment for acute coronary syndrome directed by clinicians despite improving diagnostic certainty and reducing the use of invasive coronary angiography. During the trial, we had to compromise on accepting a larger estimate for relative effect size for the trial intervention after recalculation of the sample size to account for a much lower number of primary outcome events and lower recruitment rates than anticipated. 

Several reasons could explain why the event rate was lower than the original estimate, and likely reflects the better diagnostic accuracy of newer high sensitive troponin assays and improvements in the management of patients in the past decade. Greater estimates for relative effect size have been reported in previous trials^8^ ^10^ ^11^ ^30^ although our point estimate was similar to a recent meta-analysis,^23^ which indicated a hazard ratio for subsequent myocardial infarction of 0.88 when CT coronary angiography was used in patients with acute chest pain. Although the lower confidence boundary of the primary endpoint included a clinically meaningful reduction in events, the lack of effect on treatment interventions reinforces our view that early CT coronary angiography is unlikely to influence subsequent myocardial infarction, and a larger trial with greater power would be unlikely to detect a more modest clinically meaningful effect on one year outcomes. Finally, longer term follow-up might identify further benefits in outcomes, especially if preventive treatments are more accurately targeted.

### Conclusions

Early CT coronary angiography in intermediate risk patients presenting to the emergency department with suspected acute coronary syndrome or those with a provisional diagnosis of acute coronary syndrome, had no effect on the overall treatment and prevention of the disease or one year outcomes, and was associated with an increase in the length of hospital stay. These findings do not support the routine use of early CT coronary angiography in intermediate risk patients presenting to the emergency department with acute chest pain.

**What is already known on this topic**

In stable chest pain, randomised controlled trials have shown that CT coronary angiography increases the diagnosis of coronary artery disease, resulting in more prescriptions of preventive treatments (aspirin and statins), more coronary revascularisation, and lower rates of subsequent death from coronary heart disease or non-fatal myocardial infarction
In patients presenting with acute chest pain, no definitive evidence exists that the use of CT coronary angiography affects clinical outcomes, although its use has been associated with shorter lengths of stay and increases in the use of invasive angiography and coronary revascularisation in those at very low risk
Recent international guidelines have suggested that CT coronary angiography might be considered to investigate underlying coronary artery disease in intermediate risk patients with acute chest pain although the benefits of this strategy are unknown

**What this study adds**

In intermediate risk patients with suspected acute coronary syndrome, early CT coronary angiography reduced the subsequent use of invasive angiography or testing for ischaemia compared with those receiving standard of care only
Early CT coronary angiography did not change the overall frequency of acute or preventive treatments or alter subsequent clinical events in intermediate risk patients presenting to the emergency department with acute chest pain
The findings do not support the routine use of early CT coronary angiography in all intermediate risk patients with acute chest pain

## Data Availability

Deidentified individual participant data will be made available one year after publication of the primary manuscript. Data requests should be submitted to the corresponding author.

## Web extra

Extra material supplied by authors

Web appendix: Supplementary appendix

## Notes

Contributors: AJG, CR, JES, SG, RFS, SCL, DEN, and PT contributed to the conception and design of the study. AJG, KO, RO, DEN, CR, JES, SG, RFS, NC, LK, AK, DF, SCL, and RJL were involved in the acquisition of data and in the management of the study. SCL and RJL have accessed and verified the complete study data. All authors had access to the final statistical report and contributed to analysis or interpretation of the data or both. AJG and DEN wrote the first draft of the manuscript. All authors critically revised the manuscript and agreed the manuscript to be submitted. This manuscript was written by the writing committee who had full access to the clinical trial report and vouch for the accuracy and completeness of all data and for the fidelity of the trial to the protocol. The corresponding author (AJG) attests that all listed authors meet authorship criteria and that no others meeting the criteria have been omitted.

The lead author affirms that the manuscript is an honest, accurate, and transparent account of the study being reported; that no important aspects of the study have been omitted; and that any discrepancies from the study as planned have been explained.

Dissemination to participants and related patient and public communities: We will make a plain language trial summary available to patients via trial and investigator websites, disseminate on social media platforms, and send to appropriate charity and patient organisations.

Provenance and peer review: Not commissioned; externally peer reviewed.
