# Supplementary material for: Early computed tomography coronary angiography in patients with suspected acute coronary syndrome: randomised controlled trial
Source: BMJ. 2021 Sep 29;374:n2106. doi: 10.1136/bmj.n2106 (PMC8479591; doi:10.1136/bmj.n2106)
Supplement: Supplementary file 1 — Web appendix: Supplementary appendix [file graa066318.ww.pdf]

SUPPLEMENTARY APPENDIX

Early CT Coronary Angiography in Patients with Suspected Acute Coronary Syndrome

A randomised controlled trial

Alasdair J Gray,[1,2]* Carl Roobottom,[3] Jason E Smith,[3] Steve Goodacre,[4] Katherine Oatey,[1]
Rachel O'Brien,[2] Robert F Storey,[4] Nick Curzen,[5] Liza Keating,[6] Attila Kardos,[7]
Dirk Felmeden,[8] Robert J Lee,[1] Praveen Thokala,[4] Steff C Lewis,[1] David E Newby[1,2]
on behalf of the RAPID-CTCA Investigators.

[1] University of Edinburgh, [2] Royal Infirmary of Edinburgh, [3] University Hospitals Plymouth NHS Trust, [4] University of Sheffield, [5] University of Southampton, [6] Royal Berkshire NHS Foundation Trust, [7] Milton Keynes University Hospital NHS Foundation Trust, [8] Torbay and South Devon NHS Foundation Trust.

Corresponding Author:

Name:	Professor Alasdair J. Gray
Address:	Acute Care Edinburgh (ACE)<br>Centre for Population and Health Sciences<br>Usher Institute, University of Edinburgh
Email:	alasdair.gray@ed.ac.uk
Telephone:	0131 242 3867

Sponsor
The University of Edinburgh and NHS Lothian Health Board were co-sponsors.

Trial Registration
ISRCTN19102565. Clinical Trials: NCT02284191

Keywords
Coronary heart disease, computed tomography, angina pectoris, emergency department.

Contents

**RAPID-CTCA Investigators**	**Pages 3-7**
**Supplementary Figures**	**Page 8-9**
**Figure S1**	**Page 8**
**Figure S2**	**Page 9**
**Supplementary Tables**	**Pages 10-23**
**Table S1**	**Pages 10-11**
**Table S2**	**Page 12**
**Table S3**	**Pages 13-14**
**Table S4**	**Page 15-18**
**Table S5**	**Page 19**
**Table S6**	**Page 20**
**Table S7**	**Page 21**
**Table S8**	**Page 22**
**Table S9**	**Page 23**

The RAPID-CTCA Investigators

Writing Committee:
Prof Alasdair J Gray, University of Edinburgh
Prof Carl Roobottom, Derriford Hospital, University Hospitals Plymouth NHS Trust
Prof Jason E Smith, Derriford Hospital, University Hospitals Plymouth NHS Trust
Prof Steve Goodacre, University of Sheffield
Ms Katherine Oatey, University of Edinburgh
Ms Rachel O'Brien, Royal Infirmary of Edinburgh, NHS Lothian
Prof Robert F Storey, University of Sheffield
Prof Nick Curzen, University of Southampton
Dr Liza Keating, Royal Berkshire NHS Foundation Trust
Prof Attila Kardos, Milton Keynes University Hospital NHS Foundation Trust
Dr Dirk Felmeden, Torbay and South Devon NHS Foundation Trust
Mr Robert J Lee, University of Edinburgh
Prof Steff C Lewis, University of Edinburgh
Dr Praveen Thokala, University of Sheffield
Prof David E Newby, University of Edinburgh

Trial Steering Committee:
Chairperson: Prof Tim Coats, University of Leicester
Prof Alasdair Gray, University of Edinburgh
Prof Steff Lewis, University of Edinburgh
Mr Kenneth Archibald, Retired, Layperson
Mr Graham Bell, Retired, Layperson
Mr Rodney Mycock, Retired, Layperson
Dr Russell Bull, The Royal Bournemouth and Christchurch Hospitals NHS Foundation Trust
Prof Gerry McCann, University of Leicester
Dr Tarun Mittal, Royal Brompton and Harefield NHS Foundation Trust
Dr James Rudd, University of Cambridge

Data Monitoring Committee:
Chairperson: Prof Carrol Gamble, University of Liverpool
Prof Simon Carley, Manchester University NHS Foundation Trust
Prof Simon Padley, Chelsea and Westminster Hospital NHS Foundation Trust

Endpoint Adjudication Committee:
Prof Nick Mills, University of Edinburgh
Dr Anoop Shah, London School of Tropical Medicine and Hygiene
Dr Andrew Chapman, University of Edinburgh

CTCA Quality Assurance Group:
Prof Carl Roobottom, Plymouth Hospitals NHS Trust
Dr Mark Jones, Royal Infirmary of Edinburgh
Dr Fergus Perks, Royal Infirmary of Edinburgh
Dr Gareth Morgan-Hughes, Plymouth Hospitals NHS Trust
Dr Vikram Raju, Plymouth Hospitals NHS Trust
Ms Kym Luke, Plymouth Hospitals NHS Trust

Edinburgh Clinical Trials Unit (University of Edinburgh):
Ms Ruth Armstrong, Mrs Julia Boyd, Mr David Buchanan, Mrs Christine Campbell, Mr Ronnie Harkess, Mr Robert Lee, Prof Steff Lewis, Ms Lynsey Milne, Ms Lumine Na, Ms Katherine Oatey, Mr Phillip Rayson, Ms Aryelly Rodriguez, Mrs Pamela Sinclair, Dr Lorraine Smith, Mrs Michelle Stevens, Mr Tony Wackett, Mr Allan Walker and Mr Christopher White.

Principal Investigators:
Prof Alasdair Gray, Royal Infirmary of Edinburgh
Prof Steve Goodacre, Sheffield Northern General Hospital
Prof Carl Roobottom, Prof Jason Smith, Derriford Hospital, University Hospitals Plymouth NHS Trust
Dr Dirk Feldman, Torbay Hospital
Dr Andrew Kinnon, Dr Ajay Yerramasu, Victoria Hospital, Kirkcaldy
Dr Robert Huggett, Russells Hall Hospital
Dr Liza Keating, Royal Berkshire NHS Foundation Trust
Dr Sudantha Bulugahapitiya, Bradford Teaching Hospitals
Dr Jehangir Din, Royal Bournemouth Hospital
Dr Andrew Mitchell, Jersey General Hospital
Dr Anne Scott, Borders General Hospital
Dr Anna Beattie, Royal Victoria Infirmary, Newcastle
Dr Khalid Alfakih, Lewisham University Hospital
Dr Adrian Brady, Glasgow Royal Infirmary
Prof Attila Kardos, Milton Keynes University Hospital NHS Foundation Trust
Dr Hefin Jones, University Hospitals of the North Midlands
Dr Derek Connolly, Sandwell Hospital, Birmingham
Dr Ronak Rajani, Guy's and St Thomas' NHS Foundation Trust
Dr Rangasamy Muthusamy, Dr Simon Smith, Rotherham General Hospital
Dr Abdel-Rahman Saif-El-Dean, Leeds General Infirmary
Dr Ben Holloway, Queen Elizabeth Hospital Birmingham
Dr Ansuman Saha, Surrey and Sussex Hospitals
Dr Nick Curzen, University Hospital Southampton NHS Foundation Trust
Dr Matthias Schmitt, Manchester University NHS Foundation Trust
Dr Christopher Travill, Luton and Dunstable University Hospital
Dr Ceri Davies, Prof Tim Harris, Barts Health NHS Trust Royal London Hospital
Dr Ceri Davies, Prof Tim Harris, Whipps Cross University Hospital
Dr Will Roberts, Worcestershire Acute Hospitals NHS Trust
Dr Patrick Donnelly, Ulster Hospital Belfast
Dr Justin Carter, North Tees and Hartlepool NHS Trust
Dr. John Irving, Ninewells Hospital, Dundee
Dr Chris Vorwerk, Queen Alexandra Hospital, Portsmouth
Dr Ash Basu, Betsi Cadwaladr University Health Board, Wrexham
Dr Jason Dungu, Basildon and Thurrock University Hospitals NHS Foundation Trust
Dr Elisa McAlindon, Dr Sandeep Hothi, Dr David Rosewarne, Dr Arivalagan Bapusamy, The Royal Wolverhampton NHS Trust
Dr Jonathan Watt, Raigmore Hospital, Inverness
Dr Claire McGroarty, Queen Elizabeth University Hospital, Glasgow

Trial Research Team:

Royal Infirmary of Edinburgh:
Polly Black, Caroline Blackstock, Julia Grahamslaw, Mark Jones, Collette Keanie, Margaret MacLeod, Graham McKillop, Siobhan McLaughlin, David Newby, Rachel O'Brien, Miranda Odam, Fergus Perks, Alyson Phillips, Ewan Pirie, Gillian Ritchie, Kirsty Simpson, Janet Summerside, Gordon Truong, Kirsty Weston, Michelle Williams, Jennifer Wooton

Sheffield Northern General Hospital:
Sarah Bird, Peter Brown, Hridesh Chatha, Alan Fletcher, Catherine Hill, Shery Mofidi, Hasan Qayyum, Rob Storey, Judith Sugden, Anna Wilson

Plymouth Derriford Hospital:
Alison Jeffrey, Memory Mwadeyi, Rosalyn Squire, Peter Wafer

Torbay Hospital:
Dr Lesley Archer, Lisa Felmeden, Dr Guy Gribbin, Dr Sarah Harrison, Debbie Hughes, Dr Philip Keeling, Dr Ian Mahy, Allison Summerhayes, Justine Sutton, Dr Abdullah Yonis

Victoria Hospital, Kirkaldy:
Susan Fowler, Amanda McGregor, Karen Grey, Dr Tom Hartley, Dr David Szapiro, Lorraine Dinnel, Dennis Sandeman

Russells Hall Hospital:
Julie Dean, Amy Pugh

Royal Berkshire NHS Foundation Trust:
Parminder Bhuie, Dr James Briggs, Claire Burnett, Abby Gandy, Nicola Jacques, Sarah MacGill, Dr Archie Speirs, Niamh Tolan

Bradford Teaching Hospitals:
Craig Atkinson, Dr Mark Kon, Carita Krannila, Manitha Thomas

Royal Bournemouth Hospital:
Dr Russell Bull, Stephanie Horler, Nicki Lakeman, Jane McLeod, Sara Nix, Dr Sue Thomas

Jersey General Hospital:
Dr Daniel Ahlert, Dr Christopher Edmond, Dr Christopher Hare, Kelly Anne Kinsella, Dr Jessica Langtree, Dr James Speakman, Dr Ranjit Thomas

Borders General Hospital:
Gillian Donaldson, Fiona Hall, Terry Fairbairn, Christopher Rofe

Royal Victoria Infirmary:
Jennifer Adams-Hall, Ange Bailey, Dr Kris Bailey, Leslie Bremner, Dr Ifti Haq, Angela Phillipson.

Lewisham University Hospital:
Saroj David, Osman Najam, Samia Pilgrim

Glasgow Royal Infirmary:
Claire Adams, Ammani Brown, Andrew Dougherty, Ailsa Geddes, Karen Lang, David Lowe, Ross MacDuff, Lorraine McGregor, Giles Roditi, Susan Thornton, Joyce Triscott.

Milton Keynes Hospital NHS Foundation Trust:
Felicia Adjei, Antoanela Colda, Caitlin Chapman, Veronica Edgell, Michael Fell, Laszlo Halmai, Aarzoo Khan, John Northfield, Cheryl Padilla-Harris, Mike Pashler, Gill Richie, Diane Scaletta, Sarah–Beth Sunderland, Joanne Turner, Lois Vickery, Sonya Walia, Felicity Williams, Lynn Wren, Nicola Wright

University Hospitals of the North Midlands:
Holly Maguire, Resti Varquez

Sandwell Hospitals:
Anthony D'Sa, Vinoda Sharma, Ashley Turner

Guy's and St Thomas' NHS Foundation Trust:
Megan Bell, Dr Giulia Benedetti, Kirsty Gibson, Dr Sze Mun Mak, Dr Rebecca Preston, Amy Raynsford, Ruth Sanchez-Vidal

Rotherham General Hospital:
Susan Biggins, Kathryn Dixon, Peter Kraut, Mwada Lawan, Victoria Murray, Dr Tom Mwambingu, Rachel Walker, Carol Weston.

Leeds General Infirmary:
Roo Byrom-Goulthorp, Dr Michael Darby, Dr Annette Johnstone, Eunice Ikongo, Alan Lin, Melanie Mcginlay

Queen Elizabeth Hospital Birmingham:
Tania Albutt, Vicky Dawson , Claire Dowling, Karen Isaacs, Cheyanne Kaila, Dr Gareth Lewis, Nicky Mortimer, Sunitha San, Kelly Tabor, Kealy Wright

Surrey and Sussex Hospitals:
Dr Riaz Ahmed, Sally Collins, Sarah Davies, Nokukhanya Ndlovu.

University Hospital Southampton NHS Foundation Trust:
Ausami Abbas, Alison Calver, Simon Corbett, Peter Cowburn, Andrew Flett, Huon Gray, Stephen Harden, Paul Haydock, Michael Mahmoudi, John Paisey, Charles Peebles, Drew Rakhit, John Rawlins, Paul Roberts, Benoy Shah, James Shambrook, Iain Simpson, Rohit Sirohi, Wagas Ullah, Katharine Vedwan, James Wilkinson, Arthur Yue

Manchester University NHS Foundation Trust:
Sarra Giannopoulou, Melanie Greaves, Stephen McGlynn, Chris Miller, Akhila Muthuswamy, Lindsay Murray, Anie Nicholas, Matthias Schmitt

Luton and Dunstable University Hospital:
Susan Gent, Nafisa Hussain

Barts Health NHS Trust Royal London Hospital and Whipps Cross University Hospital:
Raine Astin-Chamberlain, Ben Bloom, Olivia Bolton, Dan Martin, Lyrics Noba, Georgia Norman, Shelley Page, Helen Power, Imogen Skene, David Smith, Jon Walters

Worcestershire Acute Hospitals NHS Trust:
Angela Doughty, Elaine Byng-Hollander, Dr Helen Routledge.

Ulster Hospital Belfast:
Leah Hammond, Jayne Hutchinson, Stephanie Kelly, Susan Regan, Aileen Smith

North Tees and Hartlepool NHS Trust:
Julie Gray, Sarah Purvis, Pam Race

Ninewells Hospital:
Christine Almaden-Boyle, Kim Bissett, Carol Blues, Jackie Duff, Scot Dundas, Shirley Fawcett, Dr Graeme Houston, Emma Hutchison, Debbie Letham, Ann Mackintosh, Laura Meach, Laura Jayne Queripcz, Alan Webster

Queen Alexandra Hospital:
Julian Atchley, Zoe Daly, Kat Ellinor.

Betsi Cadwaladr University Health Board:
Richard Cowell, Helen Craddock, Rachel Hughes, Lynda Sackett, Victoria Saul, Fiona Smith, Jane Stockport, Clare Watkins

Basildon and Thurrock University Hospitals NHS Foundation Trust:
Edward Barden, Jackie Colnet, Swamy Gedeza, Laura Hoskin, Lauren Kittridge, Gracie Maloney, Claire McCormick, Anne Nicholson, Stacey Pepper, Joanne Riches, Annaliza Sevillano

The Royal Wolverhampton NHS Trust:
Vincent Amoah, Stacey Aulton, Dr Arivalagan Bapusamy, Victoria Cottam, Stella Metherell, Sarah Milgate, Elizabeth Radford, Dr David Rosewarne, Andy Smallwood

Raigmore Hospital:
Charlotte Barr, Jonathan Broadie, David Eason, Ing-Marie Logie, Debbie McDonald, Laura O'Keeffe, Donna Patience, Lesley Patience

Queen Elizabeth University Hospital:
Dr Faheem Ahmad, Nicola Baxter, Ammani Brown, Dr John Byrne, Dr Damien Collison, Tracey Hopkins, Hayley King, Dr David Lowe, Evonne McLennan, Dr Giles Roditi, Dr David Stobo, Mark Wilson, Rosie Woodward

Figure S1

Time from randomisation to CT coronary angiography

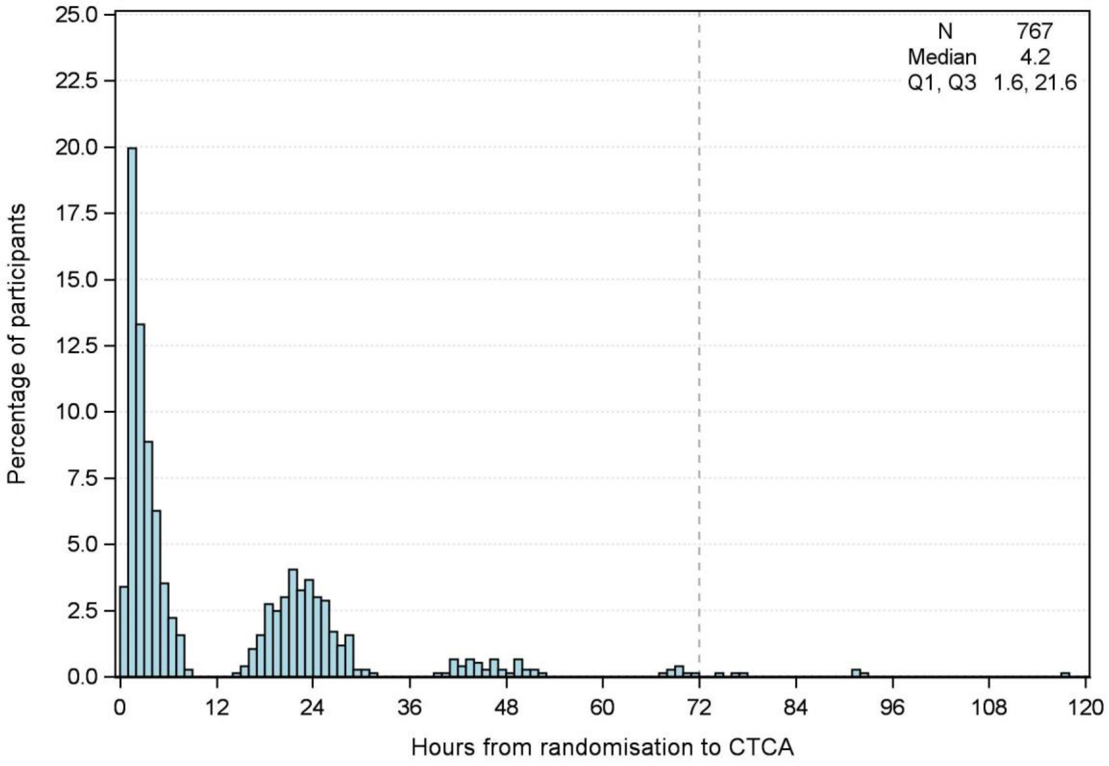

Figure S2.

Cumulative incidence of representation or recurrent admission to hospital with suspected acute coronary syndrome or recurrent chest pain up to one year

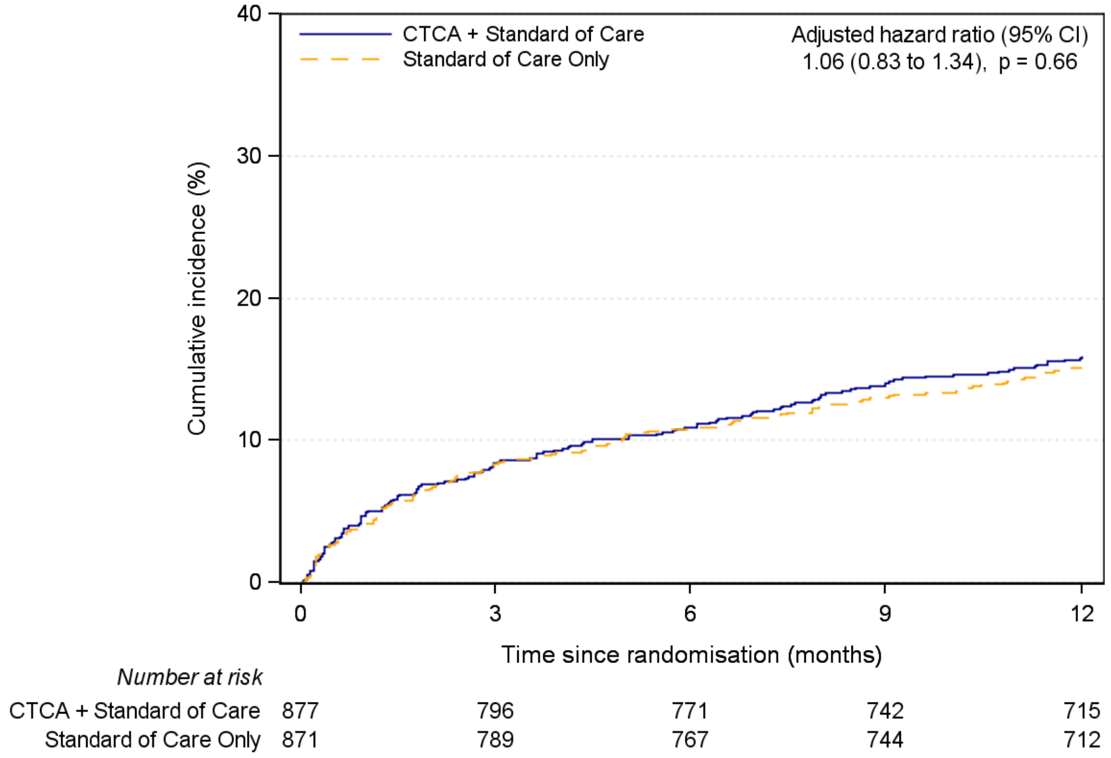

Table S1.

Trial inclusion and exclusion criteria.

Inclusion Criteria

Patient ≥18 years with symptoms mandating investigation for suspected or confirmed acute coronary syndrome with at least one of:

1. ECG abnormalities e.g. ST segment depression >0.5 mm;
2. History of ischaemic heart disease (where the clinician assessing patient confirms history based on patient history or available records);
3. Troponin elevation above the 99th centile of the normal reference range or increase in high sensitivity troponin meeting European Society of Cardiology criteria for 'rule-in' of myocardial infarction. Troponin assays varied from site to site; local laboratory reference standards were used.

Exclusion Criteria*

1. Signs, symptoms, or investigations supporting high-risk acute coronary syndrome: ST elevation myocardial infarction; acute coronary syndrome with signs or symptoms of acute heart failure or circulatory shock; Crescendo episodes of typical anginal pain; Marked or dynamic ECG changes e.g. ST depression of >3 mm; Clinical team have scheduled early invasive coronary angiography on day of trial eligibility assessment.
2. Patient inability to undergo CT: Severe renal failure (serum creatinine >250 µmol/L or estimated glomerular filtration rate <30 mL/min/1.73 m^2); Contrast allergy; Beta blocker intolerance (if no alternative heart rate limiting agent available/suitable) or allergy; Inability to breath hold; Atrial fibrillation (where mean heart rate is anticipated to be greater than 75/min after beta blockade);

3. Invasive coronary angiography or CTCA proven obstructive coronary artery disease within last 2, or normal coronary arteries within the last 5 years.
4. Previous recruitment to the trial;
5. Known pregnancy or currently breast feeding;
6. Inability to consent;
7. Further investigation for acute coronary syndrome would not in the patient’s interest, due to limited life expectancy, quality of life or functional status;
8. Prisoners.

***** During trial conduct, a further criterion was added to exclude patients where the attending clinician decided that emergent invasive angiography was required as well as increasing the time window for recruitment from hospital presentation to randomisation (from 18 hours to 24 hours).

Table S2.

Treatment guidance based on the CT coronary angiogram (CTCA).

CTCA result	Troponin result	Trial treatment recommendation
Obstructive Disease:<br>Stenosis ≥70%	Positive or Negative	1. ACS and secondary preventative therapies;<br>2. Invasive coronary angiography ± revascularisation.
Moderate Non-obstructive Disease:<br>Stenosis 50-69%	Positive	1. ACS and secondary preventative therapies;<br>2. Consider invasive coronary angiography if uncertainty about the presence of obstructive coronary artery disease or functional testing.
Moderate Non-obstructive Disease:<br>Stenosis 50-69%	Negative	1. Secondary preventative therapies;<br>2. Consider invasive coronary angiography if uncertainty about the presence of obstructive coronary artery disease or functional testing.
Mild Non-obstructive Disease:<br>Stenosis <50%	Positive	1. Consider ACS and secondary preventative therapies;<br>2. Consider alternative cause of chest pain and troponin rise.
Mild Non-obstructive Disease:<br>Stenosis <50%	Negative	1. Discharge with no further follow up;<br>2. Consider secondary preventative therapies.
Normal (no evidence of CAD)		Discharge with no further follow up.

ACS, acute coronary syndrome; CAD, coronary artery disease.

Table S3.

Trial outcomes

Primary Endpoint

The primary endpoint was all-cause death or subsequent non-fatal type 1 or type 4b myocardial infarction at one year, measured as time to first such event. Myocardial infarction was defined according to the third Universal Definition of Myocardial Infarction[13] and endpoints were adjudicated by two independent cardiologists blinded to the intervention with a third arbitrating where there was disagreement.

Key secondary endpoints up to one year

1. Coronary heart disease death or subsequent non-fatal myocardial infarction;

2. Cardiovascular disease death or subsequent non-fatal myocardial infarction;

3. Subsequent non-fatal myocardial infarction;

4. Coronary heart disease death;

5. Cardiovascular death;

6. All-cause death.

Other Secondary Endpoints up to one year unless otherwise stated

- Coronary heart disease death or subsequent non-fatal myocardial infarction (type 1 or 4b);
- Subsequent non-fatal myocardial infarction (type 1 or 4b);
- Non-cardiovascular death;
- Invasive coronary angiography;
- Coronary revascularisation;

- Percutaneous coronary intervention;
- Coronary artery bypass graft surgery;
- Proportion of patients prescribed acute coronary syndrome therapies during index hospitalisation;
- Proportion of patients discharged on preventative treatment or have alteration in dosage of preventative treatment during index hospitalisation;
- Length of stay for index hospitalisation;
- Representation or rehospitalisation with suspected acute coronary syndrome/recurrent chest pain within 12 months after index hospitalisation;
- Chest pain symptoms up to 12 months;
- Patient satisfaction at 1 month;
- Clinician certainty of presenting diagnosis after CTCA;
- Quality of life, measured by EQ-5D-5L up to 12 months.

Adverse Events and Serious Adverse Events:

- Proportion of patients with alternative cardiovascular diagnoses identified on CTCA;
- Proportion of patients with non-cardiovascular diagnosis identified on CTCA;
- Radiation exposure from CTCA as trial intervention.

* The final change to the trial outcomes are detailed in RAPID-CTCA Protocol version 7 (24/02/2020).

Table S4.

Summary of protocol changes during the trial

Version No	Date	Summary of changes made to the protocol	Date implemented
7	24/02/2020	Updates to primary and secondary endpoints:<br><br>All decisions made by members of the study team who were blinded to results-to-date split by randomised treatment.<br><br>Minor update to the primary objective/endpoint with rewording to state 'subsequent' MI instead of 'recurrent' MI as participants will not necessary have had a previous MI so this wording was inaccurate.<br><br>The secondary endpoints have been clarified to more closely reflect the planned analysis. These have been aligned to provide synergy with secondary endpoints reported for other studies of CTCA in chest pain.<br><br>'Key secondary endpoints' have been listed and these will be analysed hierarchically - only moving to the next endpoint if a significant p-value is seen. 'Other endpoints' are then listed which will all be individually analysed.<br><br>Addition of CTCA scan research repository. Updates to Statistics and Health Economics sections to better reflect SAP and HEAP.	19/06/2020
6	19/07/2018	Changes to sample size and power calculation to reflect extension request.	19/09/2018
5	17/10/2016	Update to allow 24 hours, rather than 18 hours to randomise participants.<br><br>CTCA delivery section re-worded to clarify the deviation/violation reporting process when high radiation doses are identified.<br><br>QA Reporting - update to 'experts independent to the trial site'.<br><br>Updated section 6 and 9.2.1 to include 'alternative heart rate limiting medication (instead of beta blocker)'.	25/11/2016
4	23/05/2016	Primary/secondary endpoints clarified to better reflect proposed statistical analysis:	29/06/2016

		All decisions made by members of the study team who were blinded to results-to-date split by randomised treatment.<br><br>Clarification of primary endpoint with update from "Primary end-point will be one-year all-cause death or recurrent type 1 or type 4b MI at one year and time to such event" to "Primary end-point will be all-cause death or recurrent non-fatal type 1 or type 4b MI at one year measured as time to first such event."<br><br>Secondary endpoint updated from "Proportion of patients prescribed ACS therapies and/or discharged on secondary prevention treatment or have alteration in dosage of secondary preventive treatment during index hospitalisation" and instead split into two endpoints "Proportion of patients prescribed ACS therapies during index hospitalisation" and "Proportion of patients discharged on prevention treatment or have alteration in dosage of prevention treatment during index hospitalisation.<br><br>Updated number of sites to ~35 rather than 30.<br><br>Clarification now a deviation if participants randomised to have a CTCA do not receive the scan within 72 hours.<br><br>Clarification to ambulatory scan process.<br><br>CTCA radiation dose protocol deviations now recorded/reported in DLP not mSV.<br><br>Update of Management Guidelines to include functional testing as a suggested treatment.	
3	13/11/2015	Update to primary and secondary endpoints for planned statistical analysis of the study.<br><br>All decisions made by members of the study team who were blinded to results-to-date split by randomised treatment.<br><br>Primary endpoint updated from "The primary end-point will be all-cause death or recurrent non-fatal type 1 or type 4b myocardial infarction." to "The primary end-point will be all-cause death or recurrent non-fatal type 1 or type 4b MI at one year and time to first such event."<br><br>Secondary endpoint updated from "Proportion of patients prescribed ACS therapies and/or discharged on secondary prevention treatment during index	15/01/2016

		hospitalisation" to "Proportion of patients prescribed ACS therapies and/or discharged on secondary prevention treatment or have alteration in dosage of secondary preventive treatment during index hospitalisation"<br><br>"Proportion of patients with alternative e.g. aortic dissection or incidental but potentially concerning e.g. malignancy or pulmonary nodules" updated to two separate endpoints: "Proportion of patients with alternative diagnoses that relates to presentation on CTCA e.g. aortic dissection" and "Proportion of patients with incidental finding but potentially concerning on CTCA e.g. malignancy or pulmonary nodules"<br><br>"Total average radiation exposure in each arm during index hospitalisation" updated to "Total average radiation exposure from CTCA in the intervention arm during index hospitalisation."<br><br>New secondary endpoint added "Clinician certainty of presenting diagnosis after CTCA"<br><br>Inclusion/Exclusion updates: Troponin rise now included definition - increase in high sensitivity troponin meeting European Society of Cardiology criteria for 'rule-in' or myocardial infarction; Invasive coronary angiography only exclusion if on day of trial eligibility assessment; Beta blocker intolerance now only exclusion if no alternative heart rate limiting agent available. Exclusions: Atrial fibrillation now only exclusion if mean heart rate is *anticipated* to be greater than 75 beats per minute after beta blockade.<br><br>Update to clarify the timeline in which the scan can be completed.	
2	09/12/2014	Secondary endpoints clarified.<br><br>"Proportion of patients assigned to CTCA with normal or non-diagnostic imaging" updated to "Proportion of patients assigned to CTCA with normal or mild non-obstructive disease"<br><br>"Total radiation exposure in each arm" updated to "Total average radiation exposure in each arm during index hospitalisation."<br><br>Screening process clarified.	At study start

1	27/10/2014	N/A - first draft	Never used - study started on version 2

Table S5.

Discharge diagnosis for index hospitalisation

	CT Coronary Angiography + Standard of Care (N = 877) n (%)	Standard of Care Only (N = 871) n (%)	Overall (N = 1748) n (%)
NSTEMI	350 (39.9)	339 (38.9)	689 (39.4)
Chest pain - no clear diagnosis	208 (23.7)	218 (25.0)	426 (24.4)
Unstable angina	81 (9.2)	70 (8.0)	151 (8.6)
Stable angina	64 (7.3)	64 (7.3)	128 (7.3)
Musculoskeletal pain	36 (4.1)	35 (4.0)	71 (4.1)
Other gastrointestinal pain	29 (3.3)	21 (2.4)	50 (2.9)
Pericarditis/myopericarditis	20 (2.3)	28 (3.2)	48 (2.7)
Arrhythmia	19 (2.2)	20 (2.3)	39 (2.2)
Cardiomyopathy	4 (0.5)	15 (1.7)	19 (1.1)
STEMI	9 (1.0)	8 (0.9)	17 (1.0)
Oesophageal pain	9 (1.0)	7 (0.8)	16 (0.9)
Pneumonia/pleurisy	9 (1.0)	5 (0.6)	14 (0.8)
Pulmonary embolism	5 (0.6)	6 (0.7)	11 (0.6)
Heart failure	5 (0.6)	5 (0.6)	10 (0.6)
Anxiety	6 (0.7)	3 (0.3)	9 (0.5)
Syncope	3 (0.3)	5 (0.6)	8 (0.5)
Coronary artery spasm	3 (0.3)	4 (0.5)	7 (0.4)
Costochondritis	2 (0.2)	3 (0.3)	5 (0.3)
Valvular heart disease	1 (0.1)	2 (0.2)	3 (0.2)
Acute aortic syndrome	2 (0.2)	0 (0.0)	2 (0.1)
Symptomatic anaemia	0 (0.0)	2 (0.2)	2 (0.1)
Other	12 (1.4)	11 (1.3)	23 (1.3)

NSTEMI, non-ST segment elevation myocardial infarction; STEMI, ST segment elevation myocardial infarction.

Table S6.

Reasons for non-adherence with the trial intervention of computed tomography coronary angiography

	n
**Total**	**110**
CT scanner unavailable	26
Clinician decision not to proceed with scan	16
Patient deterioration	13
High coronary artery calcium score	13
Heart rate issue	13
Patient non-compliant in scan	6
Radiologist not available	6
Cannula issue	5
Patient declined scan	5
Exclusion criterion identified after randomisation [a]	4
Death	1
Other reason [b]	2

[a] Patient unable to undergo CT coronary angiogram due to allergy to contrast (n=2), eGFR too low for local radiology guidelines (n=1), or CT coronary angiogram within last 5 years (n=1).

[b] Patient transferred to another hospital before CT coronary angiogram could be completed (n=1), and CT coronary angiogram not performed as it was incorrectly thought patient had been randomised to standard of care only arm (n=1).

Table S7.

Other secondary cardiovascular outcomes

Outcome within 12 months	CT Coronary Angiography + Standard of Care (N = 877)	Standard of Care Only (N = 871)	Estimate	Hazard Ratio (95% Confidence Interval)	P-value
Coronary heart disease death or non-fatal myocardial infarction (type 1 or 4b)	43 (4·9%)	43 (4·9%)	Unadjusted	0·99 (0·65 to 1·51)	0·95
			Adjusted	0·97 (0·63 to 1·48)	0·88
Non-fatal myocardial infarction (type 1 or 4b)	35 (4·0%)	38 (4·4%)	Unadjusted	1·03 (0·69 to 1·55)	0·88
			Adjusted	1·02 (0·67 to 1·53)	0·94
Non-cardiovascular disease death	7 (0·8%)	9 (1·0%)	Unadjusted	1·03 (0·69 to 1·54)	0·88
			Adjusted	1·01 (0·68 to 1·52)	0·95

Table S8.

Chest pain symptoms reported on WHO Rose angina questionnaire

	CT Coronary Angiography + Standard of Care (N = 877) n (%)	Standard of Care Only (N = 871) n (%)
Symptoms at one month		
No chest pain	213 (34.1)	205 (35.2)
Non-exertional chest pain	180 (28.8)	152 (26.1)
Chest pain on exertion	232 (37.1)	226 (38.8)
	n = 625	n = 583
Symptoms at six months		
No chest pain	278 (45.8)	264 (47.3)
Non-exertional chest pain	142 (23.4)	119 (21.3)
Chest pain on exertion	187 (30.8)	175 (31.4)
	n = 607	n = 558
Symptoms at twelve months		
No chest pain	287 (48.9)	248 (47.2)
Non-exertional chest pain	121 (20.6)	107 (20.4)
Chest pain on exertion	179 (30.5)	170 (32.4)
	n = 587	n = 525

Table S9.

Quality of life as measured by mean utility values from EQ-5D questionnaire

	**CT Coronary Angiography + Standard of Care (N = 877)**	**Standard of Care Only (N = 871)**
Number of participants included in analysis	764	713
Baseline	0.765	0.768
One month	0.739	0.738
Six months	0.758	0.769
Twelve months	0.761	0.765

The quality of life analysis excluded the 113 participants in the CTCA + standard of care arm and 158 participants in the standard of care only arm without any follow-up utility values, i.e. they did not respond to any of the EQ-5D-5L questionnaires at one, six, and twelve months. Of the 764 participants in the CTCA + standard of care arm who responded to at least one of the EQ-5D-5L questionnaires at one, six, and twelve months there were 72, 102, and 130 who did not respond to the questionnaires at one, six, and twelve months respectively. Similarly, of the 713 participants in the standard of care only arm who responded to at least one of the EQ-5D-5L questionnaires at one, six, and twelve months there were 92, 117, and 143 who did not respond to the questionnaires at one, six, and twelve months respectively. Multiple imputation techniques were employed to impute these missing utility values at one, six, and twelve months. The imputed values were used in the calculation of the mean utility values.
